# Pathogenic Analysis of the Bronchoalveolar Lavage Fluid Samples With Pediatric Refractory *Mycoplasma pneumoniae* Pneumonia

**DOI:** 10.3389/fcimb.2020.553739

**Published:** 2020-10-28

**Authors:** Fei Zhao, Jinrong Liu, Di Xiao, Liyong Liu, Jie Gong, Juan Xu, Huimin Li, Shunying Zhao, Jianzhong Zhang

**Affiliations:** ^1^National Institute for Communicable Disease Control and Prevention, Chinese Center for Disease Control and Prevention, State Key Laboratory of Infectious Disease Prevention and Control, Collaborative Innovation Center for Diagnosis and Treatment of Infectious Diseases, Beijing, China; ^2^Department of Respiratory Medicine, Beijing Children's Hospital Affiliated to Capital Medical University, National Center for Children's Health, Beijing, China

**Keywords:** *Mycoplasma pneumoniae*, refractory *Mycoplasma pneumoniae* pneumonia, bronchoalveolar lavage fluid, co-infection, macrolide resistant

## Abstract

**Background:** We conducted a pathogenic analysis in the bronchoalveolar lavage fluid (BALF) samples from refractory *Mycoplasma pneumoniae* pneumonia (RMPP) children.

**Methods:** A total of 150 BALF samples from 60 RMPP patients were analyzed to investigate pathogenic changes. The characteristics of *M. pneumoniae* were analyzed through culture, real-time PCR, genotyping, antimicrobial susceptibility testing and proteomics. The other pathogens were determined using culture, sequencing and nucleic acid detection.

**Results:** In 60 RMPP cases, the bacterial co-infection rate was 5%, while that of virus was 33.3%. The poor prognosis rate was 61.7%. The DNA positive rate among the 150 samples was 98.7%, while the culture positive rate was 56.7% for *M. pneumoniae*. Significant differences were noticed in the positivity of *M. pneumoniae* culture obtained from samples with a disease course of at least 3 weeks compared with those within 3 weeks. The genotype 1 *M. pneumoniae* strains showed a macrolide resistant (MLr) rate of 100%, and that for genotype 2 was 90.1%. Proteomics showed that there were 57 proteins up-regulated in the MLs *M. pneumoniae*, half of which were membrane-associated protein with adhesion or toxicity.

**Conclusions:** Pediatric RMPP usually presented with viral co-infection, but it caused limited effects on the progression and prognosis of RMPP. Persistent presence of viable *M. pneumoniae* is not necessary in the later stage of RMPP. The expression of virulence factor in the MLr *M. pneumoniae* was higher than that of the MLs *M. pneumoniae*, which was more common in the RMPP children.

## Background

*Mycoplasma pneumoniae* is an important pathogen responsible for about 10%-40% of community acquired pneumonia (CAP) in children and adolescence (Dumke et al., 2014; He et al., 2016; Waites et al., 2017). Although *M. pneumoniae* pneumonia (MPP) is usually considered as a self-limited disease, it may trigger pulmonary complications, which may progress into refractory *M. pneumoniae* pneumonia (RMPP) and even lethal pneumonia (Li et al., 2016; Yan et al., 2019). In a recent survey in United States, *M. pneumoniae* is considered the most common pathogen in the hospitalized individuals aged <18 years old, and about 12% of hospitalized children with *M. pneumoniae* infection are transferred to the intensive care unit (ICU) (Kutty et al., 2019). Therefore, special attention should be paid to the management of the RMPP.

Nowadays, more and more RMPP cases have been reported especially in the east Asia (Liu et al., 2018b; Okumura et al., 2019; Zhao et al., 2019b). Compared to the common *M. pneumoniae* pneumonia (CMPP) cases, RMPP patients commonly showed prolonged fever and high C-reactive protein (CRP) in peripheral blood. Besides, they were also associated with airway hypersecretion in bronchoscopy, and high-density consolidation in chest imaging. To date, the mechanism of RMPP is still not well-defined. Previous studies showed that RMPP might be related to the reaction to the pathogens induced by excessive cell-mediated immunity and cytokines (Wang et al., 2014; Zhang et al., 2016). In 2017, Yu et al. (2017) used isobaric tags for relative and absolute quantification (iTRAQ) technique to analyze *M. pneumoniae*-related proteins in serum samples from RMPP patients, which indicated that the higher CRP and other signs in these patients were triggered by excessive immunological reaction. All these studies focused on the clinical data of RMPP patients, however, few studies have been conducted to investigate the etiology of RMPP. Little is known about the virulence and antigenicity of certain types, the link between RMPP progression and the persistent infection of *M. pneumoniae*, the presence of co-infection during the RMPP, as well as the effects of the infection course on the outcome of the patients.

In this study, a dynamic analysis was performed to the pathogens in the bronchoalveolar lavage fluid (BALF) samples collected from 60 RMPP patients. We aim to analyze the tendency of *M. pneumoniae* and other pathogens during the onset of RMPP in children. Meanwhile, we preliminary analyzed the features of *M. pneumoniae* in these patients through *M. pneumoniae* culture, DNA quantitative analysis, genotyping and antimicrobial susceptibility testing, together with the biological features of RMPP-related *M. pneumoniae* through proteomics.

## Methods

### Patients

In this retrospective study, we reviewed the medical records of 60 pediatric patients with RMPP that were admitted in the Department of Respiratory Medicine at Beijing Children's Hospital between January 2016 and March 2018. RMPP was diagnosed based on the following criteria: those with MPP showing persistent fever and deterioration of clinical and radiological findings after a 7-day or more treatment using macrolide antibiotics (Subspecialty Group of Respiratory Diseases, Society of Pediatrics, Chinese Medical Association, Editorial Board, Chinese Journal of Pediatrics, 2007; Tamura et al., 2008; Subspecialty Group of Respiratory Diseases, The Society of Pediatrics, Chinese Medical Association, Editorial Board, Chinese Journal of Pediatrics, 2013). All patients showed pneumonia-related symptoms at the early-stage including persistent fever (>38.5°C), cough, and abnormal chest imaging findings. *M. pneumoniae* infection was diagnosed based on immunoglobulin M-specific anti-MP antibody titer of ≥ 1:320, or 4-fold rise of the titer in acute and convalescent serum specimens.

All the 60 patients received at least 2 courses of bronchoscopic treatment in Beijing Children's Hospital. Patients with asthma, immunodeficiency or other chronic diseases were excluded from this study. Patient information including age and gender was collected within the first 15 days of the disease course. The white blood cells (WBCs) count was determined together with the determination of CRP concentration. Meanwhile, conventional bacterial and fungal culture of BALF were recorded. Written informed consent was obtained from the patients. The study was approved by the Ethics Committee of National Institute for Communicable Disease Control and Prevention.

### Fiberoptic Bronchoscopy and BALF Collection

BALF samples were collected using flexible fiberoptic bronchoscopy. In brief, the working tip of bronchoscope was inserted into damaged lung lobe, followed by instilling of warm sterile saline and recovery using gentle suction. Afterwards, the BALF samples (1.0 mL) from each patient were used for traditional bacterial and fungal culture within 30 min after sample collection. The remanent BALF samples were transmitted to National Institute for Communicable Disease Control and Prevention, Chinese Center for Disease Control and Prevention (ICDC, China CDC) using cold chain ways for subsequent analysis.

### Pathogen Detection of BALF Samples

#### Bacterial/Fungal Culture and Identification

BALF samples (200 μL) were subject to conventional bacterial and fungal culture in the ICDC lab. The culture with positive results underwent monoclonal passage. The purified culture was identified using the Matrix-assisted laser desorption/ionization time-of-flight mass spectrometry (MALDI-TOF MS) (Xiao et al., 2014).

#### DNA Extraction and Bacterial/Fungal Nucleic Acid Detection

DNA was extracted from each BALF sample by (200 μL) using the QIAamp DNA Mini Kit (Qiagen, Germany). The total nucleic acids (TNA) were extracted by using the AllPrep DNA/RNA/miRNA Universal Kit (Qiagen, Germany). Then the DNA samples were used to amplify the bacterial 16s rRNA and fungal Internal Transcribed Spacer (ITS) sequence according to the previous description (Baker et al., 2003; Stielow et al., 2015). The products were sequenced by Sangon Biotech (Beijing, China). Real-time PCR was performed using the TNA as the template to detect the 7 common respiratory tract viruses with the commercial kit (Applied Biological Tech, Beijing, China).

### *Mycoplasma pneumoniae* Related Detection of BALF Samples

#### *Mycoplasma pneumoniae* DNA Quantitative Analysis in BALF

The BALF sample (200 μL) was added to propidium monoazide (PMA) until a final concentration of 100 μM at room temperature, followed by incubating for 15 min in dark. Then the samples were exposed to LED light source for 10 min to separate the deactivated PMA. Subsequently, the viable *M. pneumoniae* DNA was extracted using QIAamp DNA Mini Kit. Quantitative analysis of total and viable *M. pneumoniae* DNA was carried out based on the standard curve established using the standard concentration (2 copies/μl-2 × 10^7^ copies/μl) of *M. pneumoniae* (Zhao et al., 2012a).

#### *Mycoplasma pneumoniae* Genotyping

The genotyping (i.e., genotype 1 and 2) was performed using real-time PCR assay (Zhao et al., 2015). Then multilocus genotyping was conducted using the multiple locus variable-number tandem repeat analysis (MLVA) according to the previous description (Chalker et al., 2015).

#### *Mycoplasma pneumoniae* Culture

Each BALF sample (200 μL) was inoculated on *Mycoplasma* selective medium (OXOID, Thermo Fisher, NY, USA) at 37°C. A yellow color with no turbidity was considered to be *M. pneumoniae* positive (Zhao et al., 2019a). Then 0.1 ml *M. pneumoniae* positive suspension was transferred onto agar to subculture. The *M. pneumoniae* isolates were purified using dilution technique.

#### 23S rRNA Gene Amplification

Domain V of the *23S rRNA* gene was amplified with 150 BALF DNA using the primers according to the previous study (Matsuoka et al., 2004). The amplification products were sequenced using Sanger technique by Sangon Biotech (Beijing, China).

#### Antimicrobial Susceptibility Testing

Minimum inhibitory concentrations (MICs) against four antibiotics, including erythromycin, azithromycin, levofloxacin and tetracycline (Sigma-Aldrich, California, CA, United States), were determined using SP4 broth (Remel) based on the micro-dilution methods. CSLI M43-A (2017 version) was used for the MIC test.

### Proteomics Analysis of *M. pneumoniae*

#### Whole Cell Protein Preparation

Four representative *M. pneumoniae* strains were selected for the proteomics analysis. Among these strains, two were obtained from RMPP cases including Case 45 with genotype 2 (the only strain of macrolide susceptible, MLs) and Case 35 with genotype 2 (macrolide resistant, MLr). The other two strains were derived from the ICDC P033 (MLs, genotype 2) and ICDC 21109 (MLr, genotype 2) that were separated from the CMPP pediatric patients. Then differential proteomics analysis was conducted between Case 45 and ICDC 21109, and between Case 35 and ICDC P033. Four strains were incubated in cell culture flask (150 ml) at 37°C for 7 days. At the time of harvest, the cells and spent media were poured into 50 ml polycarbonate tubes and centrifuged at 10,000 g for 20 min. The pellets were subject to protein extraction using 8 M urea containing 50 mM triethyl ammonium bicarbonate (TEAB). Finally, the protein concentration was determined using BCA Protein Assay Kit (Thermo-Fisher Scientific).

#### Trypsin Digestions and Peptides Purification

The proteins were reduced by incubation with TCEP (200 mM) at 55°C for 1 h and alkylated by incubation with iodoacetamide (IAA, 375 mM, Thermo Scientific) for 30 min in dark at room temperature. TEAB (100 mM) was used to adjust the urea concentration of <1M in all the protein samples, and then the proteins were digested to peptides using trypsin (Promega) at a trypsin/protein ratio of 1:50 (w/w) overnight at 37°C. The generated tryptic peptides were dried by speed vacuum at 4°C and desalted with C18 Spin column.

#### Nano-HPLC-MS/MS Analysis

The samples were reconstituted in 0.1% formic acid (FA) and separated on a NanoAcquity Ultra Performance Liquid Chromatography (UPLC) system (EASY-nLC 1000, Thermo Scientific). Afterwards, the samples were fitted with a nanoAcquity Symmetry C18 trap column (100 μm × 2 cm, NanoViper C18, 5 μm, 100Å) and a analytical column (75 μm × 15 cm, NanoViper C18, 3 μm, 100Å). The mobile phase A was 100:0.1 HPLC grade water/FA, and mobile phase B was 100:0.1 ACN/FA. Each sample was loaded on the trapping column with a flow rate of 2.0 μL/min, followed by separation on the analytical column using a 100 min 3–35% mobile phase B linear gradient at a flow rate of 0.8 μL/min. Retention Time Calibration Mixture (Thermo Scientific) was used to optimize LC and MS parameters and was used to monitor the stability of the system.

The analytical column was coupled to a high-resolution Q-Exactive Plus mass spectrometer (Thermo Fisher Scientific, San Jose, CA) using a nano-electrospray ion source, which was operated in positive ion mode. The source was operated at 2.0 kV with transfer-capillary temperature maintained at 250°C and S-Lens RF level set at 60. MS spectra were obtained by scanning over the range m/z 350–2000. Mass spectra was acquired in the Orbitrap mass analyzer with 1 microscan per spectrum for both MS and MS/MS. Resolving power for MS and MS/MS was set at 70,000 and 17,500, respectively. Tandem MS data were acquired in parallel with MS, on the top 20 most abundant multiply charged precursors, with higher energy collisional dissociation (HCD) at normalized collision energy of 30 V. Precursors were isolated using a 2.0 m/z window and dynamic exclusion of 60 s was enabled during precursor selection. The data were determined at least twice.

#### Proteome Data Analysis

Proteome Discoverer (version 1.4, Thermo Scientific, USA) was used to search the UniProtKB/Swiss-Prot database. The parameters were set as follows: integration tolerance, 20 ppm; precursor mass tolerance, 10 ppm; fragment mass tolerance, 0.02 Da. Dynamic modification Oxidation/+15.99 Da and carbamidomethyl/+57.02 Da) were set as dynamic and static modifications. Differentially expressed proteins were determined by peptide identifications with 95% confidence interval. Meanwhile, TMT signal analyses showed at least two-fold change in abundance.

### Statistical Analysis

The data were entered into Excel 2007 sheet. SPSS 17.0 software was utilized for the statistical analysis. Student's *t*-test was utilized for the comparison between CPR values in the single RMPP cases and the co-infection RMPP cases, as well as the quantitative comparison between total and viable *M. pneumoniae* DNA in BALF samples. Chi square test was used to compare the outcome between the single RMPP cases and those with co-infection, as well as the *M. pneumoniae* cultural positivity rate of BALF samples in different stages of RMPP conditions. *P*-value of less than 0.05 was considered to be statistically significant.

## Results

### Clinical Characteristics and Prognosis of Patients

**Figure 1:**
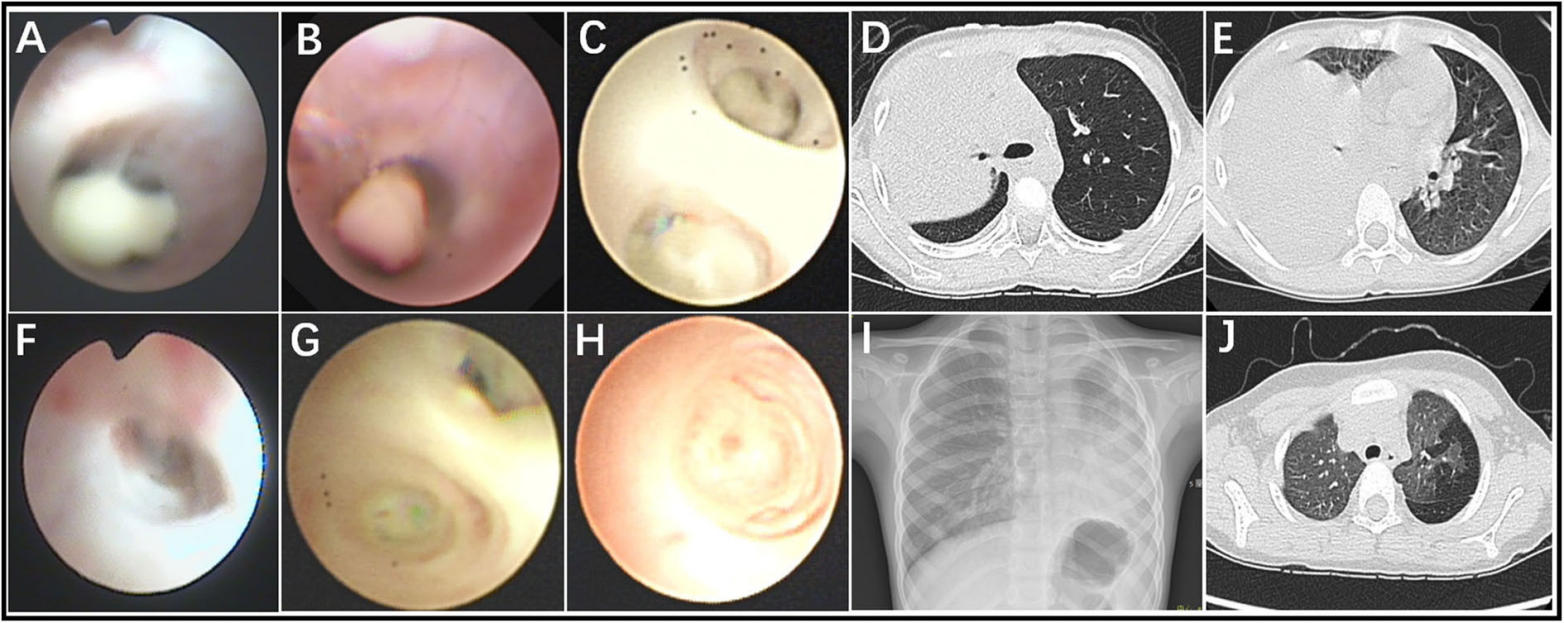
Fiberoptic bronchoscopy revealed mucous plug **(A–C)**, airway stenosis **(F)**, and airway obliteration **(G,H)**, which suggested airway hyper secretion or airway obstruction in RMPP patients. Chest imaging revealed high-density consolidation on early stage **(D,E)** and bronchitis/bronchiolitis obliterans **(I,J)** on late stage in RMPP patients.

**Figure 2:**
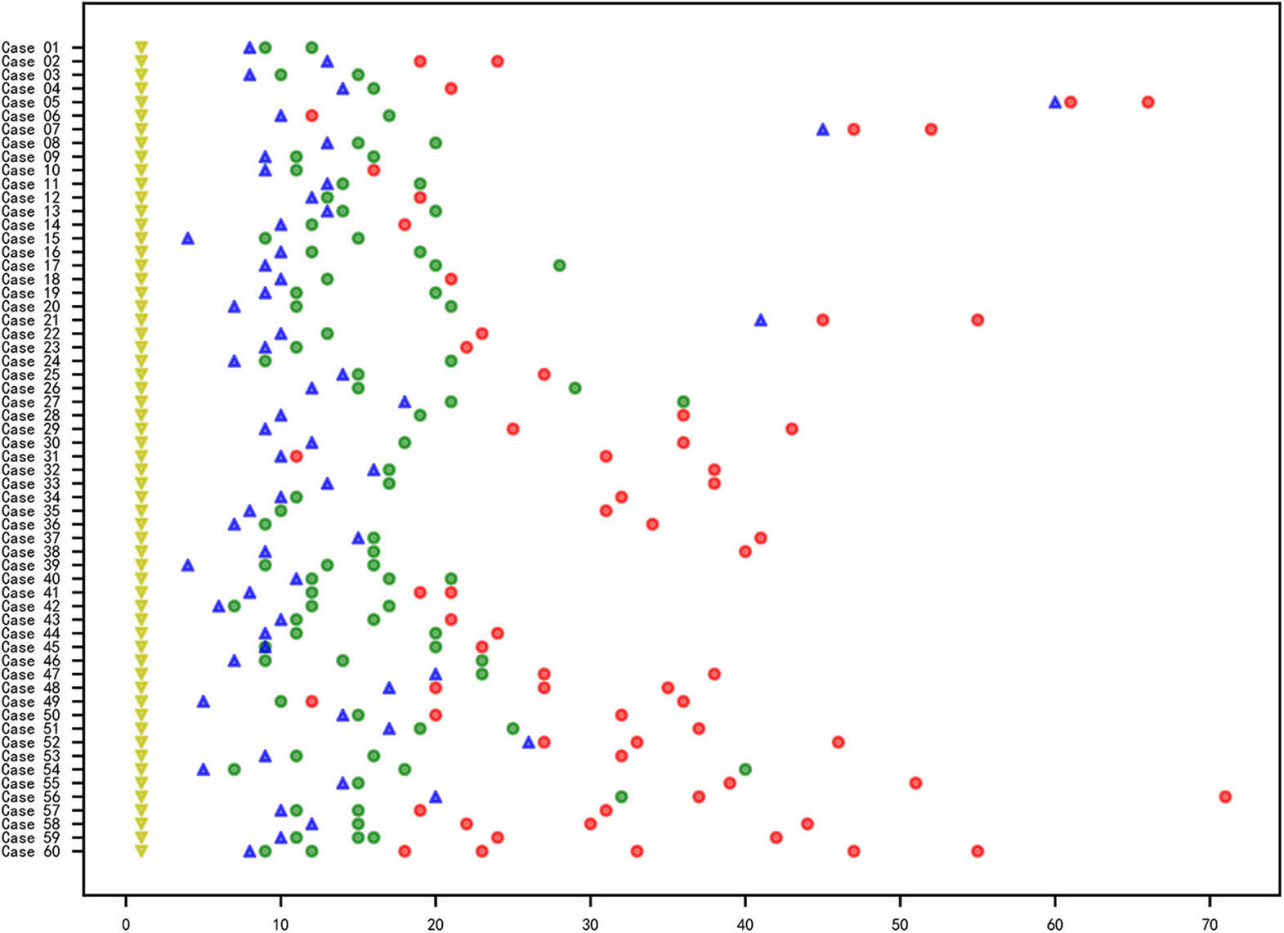
Information of the RMPP course, sample collection and *M. pneumoniae* culture of the 60 cases. X axis represented for the day and the Y axis represented for the case No. The yellow triangle (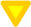): first onset of disease; blue triangle (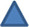): time point for the admission to Beijing Children's Hospital affiliated to Capital Medical University; circle: collection time point for the BALF samples; red circle (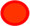): negative for *M. pneumoniae* culture; green circle (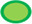) positive for *M. pneumoniae* culture.

In total, 60 RMPP patients (male: 27; female: 33; age: 3 years 3 months to 13 years 6 months; mean age: 6 years 10 months) were enrolled in our study. All the disease course of these patients upon admission to our hospital was recorded, together with the BALF collection time points. Before admission, the majority of cases received treatment in the other hospitals using ML antibiotics (e.g., Azithromycin). All patients showed typical symptoms related to airway inflammation and infection such as mucus plug or airway stenosis, or even obliteration in bronchoscopy (Figures 1A–C,F–H) and high-density consolidation in chest imaging (Figures 1D,E). Some of them developed sequelae such as bronchiolitis/bronchitis obliterans due to airway remodeling (Figures 1I,J). On this basis, they had to receive multiple bronchoscopy lavage therapy and even bronchoscopic interventional therapy. Among these patients, 38 cases (Case 01–38) received BALF sample collection twice, while 18 cases (Case 39–56) received BALF sample collection thrice. Four cases (Case 57–60) received BALF sample collection at least four times (Figure 2). The averaged WBC counts and CRP (the maximum value within the first 15 days of disease course of each patient was used for calculation of the mean) of all patients were (11.76 ± 3.73) × 10^9^/L and 107.38 ± 81.17 mg/L, respectively. The last bronchoscopy was performed on a disease course of 12–77 (median: 28.5 days) for the 60 patients. After treatment, 23 cases showed good outcome, while the rest 37 cases showed various sequela that was featured by bronchial stenosis or occlusion, sub-bronchial stenosis or occlusion, as well as pulmonary embolism.

### Co-infection of BALF in Pediatric RMPP Patients

Among the 150 BALF samples, two showed positivity for the bacterial culture in the hospital, and two showed positivity in the bacterial culture in the ICDC laboratory. Negativity was obtained for the fungal culture in the hospital and ICDC, respectively. The 16s rRNA detection results indicated that three samples were positive for bacteria, and no fungus were identified with ITS sequences. Real-Time PCR result indicated that 26 samples obtained from 20 cases were positive for viral detection. Five cases were confirmed to be positive for at least two viruses (Table 1). The mean CRP value in the single RMPP patients showed no statistical differences compared with that of the RMPP patients with co-infection (103.5 ± 66.86 vs. 19.33 ± 87.38, *t* = −0.258, *P* > 0.05). The poor prognosis rate of patients with co-infection showed no statistical differences compared with those with single RMPP (60% vs. 62.5%, χ^2^ = 0.035, *P* > 0.05, Table 1).

**Table 1:** Clinical and laboratory data of the BALF samples in 60 RMPP patients with single *M. pneumoniae* infection and co-infection.

**Group**	**Number**	**Pathogen for co-infection**	**Patient No. (the No. after the “-” represented the sampling times)**	**CRP (mg/L)**	**Rate of poor prognosis ^**d**^ (%)**
Co-infection group	20	Rothia dentocariosus	Case03-1 ^b^^,^^c^	103.5 ± 66	60.0
		Streptococcus pneumoniae	Case44-1 ^a^^,^^b^^,^^c^, Case51-2 ^c^		
		Respiratory syncytial viruses	Case01-1, Case01-2, Case06-1, Case06-2, Case41-1, Case45-1, Case60-5		
		Adenovirus	Case14-1, Case32-2, Case44-1, Case49-1, Case55-2,		
		Influenza A virus	Case04-1, Case28-2, Case49-1,		
		Influenza B virus	Case32-2, Case38-1, Case43-1,		
		Parainfluenza virus, type I	Case04-1, Case04-2, Case12-1, Case12-2, Case36-2, Case58-3, Case60-2,		
		Parainfluenza virus, type III	Case50-2, Case55-1		
Single *M. pneumoniae* infection	40	None	The residual 122 samples	109.33 ± 87.38	62.5

a Culture and identification results in the hospitals;

b Culture and identification results in the laboratories;

c *The 16s rRNA amplification sequence results for the laboratory samples*.

d *Poor prognosis was mainly featured by airway stenosis and closure (part of the children showed pulmonary embolism)*.

### *Mycoplasma pneumoniae* Culture and DNA Quantitation

**Figure 3:**
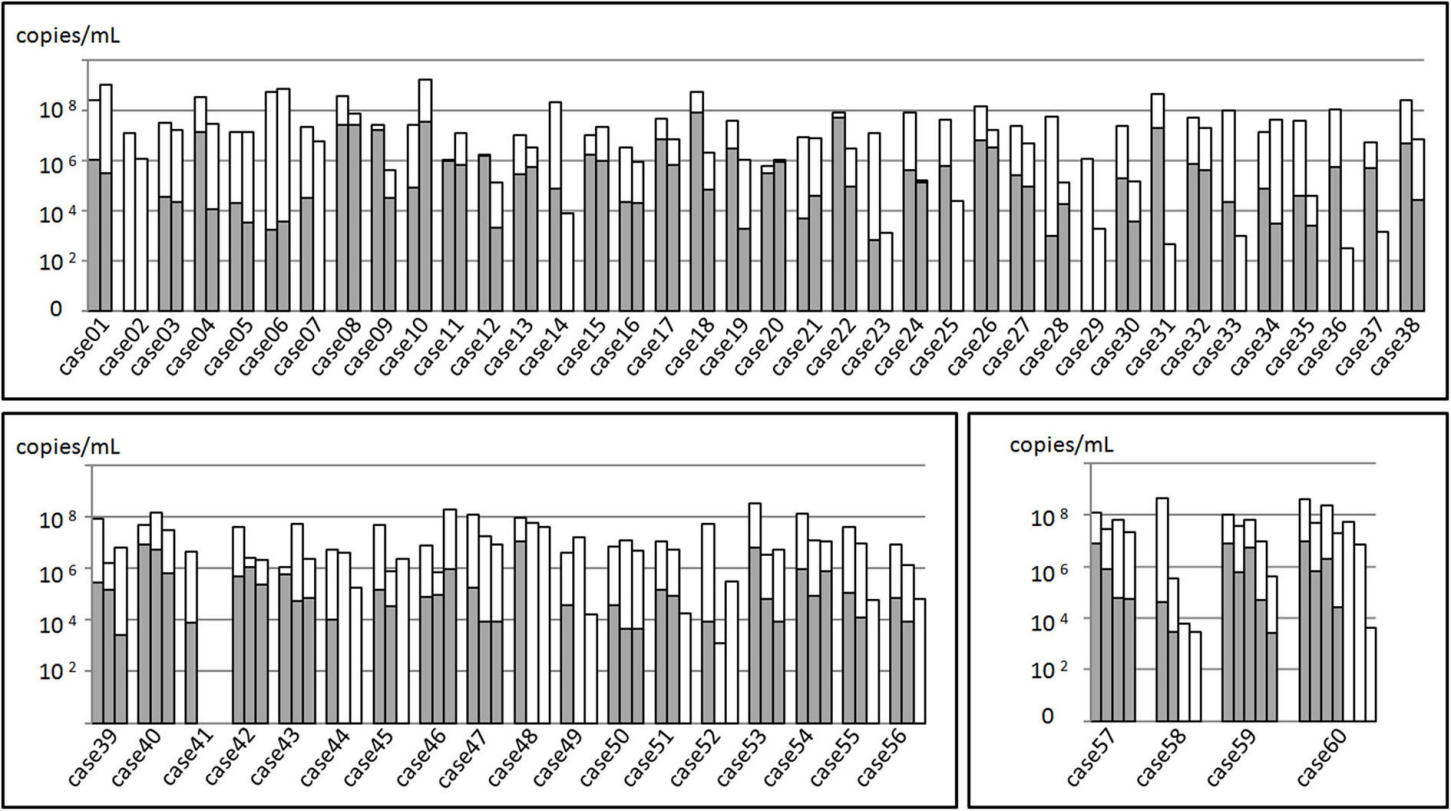
*Mycoplasma pneumoniae* DNA quantitation analysis of 150 BALF samples from 60 patients. The total height of the column including the white and gray parts represented the total *M. pneumoniae* DNA load of the samples. Gray part represented the DNA load of viable *M. pneumoniae*. In the upper part, 38 patients received sampling twice, while in the left inferior part, 18 received sampling thrice. In the right inferior part, 4 received at least three sampling.

The total samples *M. pneumoniae* culture positive rate was 56.7% (85/150), while the sample culture positive rate within 3 weeks after disease onset was 82.8%, which was far higher than that of the 3-week positive rate (82.8% vs. 14.0%, χ^2^ = 68.045, *P* < 0.05, Figure 2). All the samples were *M. pneumoniae* positive with real-time PCR detection by the total DNA except two were negative (Case 41-2 and Case 41-3). The total DNA load for *M. pneumoniae* positive samples was in a range of 3.4 × 10^2^-1.6 × 10^9^ copies/ml. The viable *M. pneumoniae* DNA positive rate was 79.3%, and the viable *M. pneumoniae* DNA load (PMA real-time PCR quantitative analysis for *M. pneumoniae*) was in a range of 6.7 × 10^2^-8.5 × 10^7^ copies/ml. There were statistical differences between the total and the viable *M. pneumoniae* DNA content (*t* = 4.553, *P* < 0.05). One sample (Case 44-2) that showed negative result in the PMA real-time PCR detection was confirmed to be culture positive for *M. pneumoniae*, while the other thirty samples with negative PMA real-time PCR detection were confirmed to be *M. pneumoniae* negative culture. Moreover, among the 33 samples negative in *M. pneumoniae* culture but positive in PMA real-time PCR detection, the majority (81.8%) showed a DNA load of <10^5^copies/ml (Figure 3).

### *Mycoplasma pneumoniae* Genotyping

In total, 116 samples (116/150) were classified into genotype 1, and 26 samples were classified into genotype 2, and 8 samples showed negativity results due to insufficient DNA copies or amplication inhibitors. Based on the case analysis, the *M. pneumoniae* genotype of BALF samples obtained from the same patient at different time points was consistent with no alternations. Therefore, given the analysis based on the 60 RMPP cases, the proportion of *M. pneumoniae* strains with a genotype 1 and 2 was 81.7% and 18.3%, respectively. By MLVA genotyping, 88 were classified into 4-5-7-2, 24 of 3-5-6-2, 10 of 4-5-7-3, and 2 of 4-5-5-2. The other 26 samples were incomplete or negative in the MLVA genotyping. For the same patient, the MLVA genotype of the samples collected from different time points was consistent with no alternation. Among the 60 RMPP patients, 44 (73.3%) were confirmed with a MLVA genotype of 4-5-7-2, 11 (18.3%) with a MLVA genotype of 3-5-6-2, 4 (6.7%) with a MLVA genotype of 4-5-7-3, and 1 (1.7%) with a MLVA genotype of 4-5-5-2. Among these aspects, the 4-5-7-2, 4-5-7-3 and 4-5-5-2 were genotype 1, while the 3-5-6-2 was genotype 2, respectively (Table 2).

**Table 2:** Genotype characteristics and MIC ranges of four antimicrobial agents used against 53 *M. pneumoniae* clinical isolates from RMPP patients.

**Mutation in the 23S rRNA**	**Isolates number**		**MLVA genotype (Numbers)**		**MIC (μg/ml)**			
	**Genotype 1**	**Genotype 2**	**Genotype 1**	**Genotype 2**	**ERY**	**AZM**	**LVX**	**TET**
A2063G	42	8	4/5/7/2(38) 4/5/7/3(4) 4/4/7/2(1)	3/5/6/2(8)	≥256	2–32	0.25–1	0.016–0.125
A2063T	1	0	4/5/7/2(1)	0	≥256	8	0.25	0.016
None	0	1	0	3/5/6/2(1)	≤ 0.008	≤ 0.008	0.5	0.016

*The MIC of each agent was defined as the lowest concentration of each antibiotic that prevented the color change*.

*ERY, erythromycin; AZM, azithromycin; LVX, levofloxacin; TET, tetracycline*.

### *Mycoplasma pneumoniae* Antimicrobial Susceptibility Results

For the 23s rRNA sequencing, 59 cases (98.3%) presented point mutation at 2063 site (A2063G, 58 cases; A2063T, 1 case), which was associated with resistance to the macrolide (MLr). In another 1 macrolide susceptible (MLs) strain, there was no mutation in the 23s rRNA. In the views of genotype, the genotype 1 *M. pneumoniae* showed MLr of 100%, while that for the genotype 2 was 90.9%. The MIC results for the *M. pneumoniae* strains from 52 cases were consistent with the genotyping results of 23s rRNA. No *M. pneumoniae* strains were isolated from 8 cases (i.e., Case 02, 05, 07, 21, 29, 31, 48 and 52). *M. pneumoniae* strains from 51 cases (98.1%) were resistant to the macrolide antibiotics, with a MIC value of 256–≥256 μg/ml and 2–64 μg/ml for Erythromycin and Azithromycin, respectively. Only one strains from Case 45 were susceptible to the ML. No strains with resistance to the tetracycline and levofloxacin were identified (Table 2).

### Nano-HPLC-MS/MS Proteome Analysis

**Figure 4:**
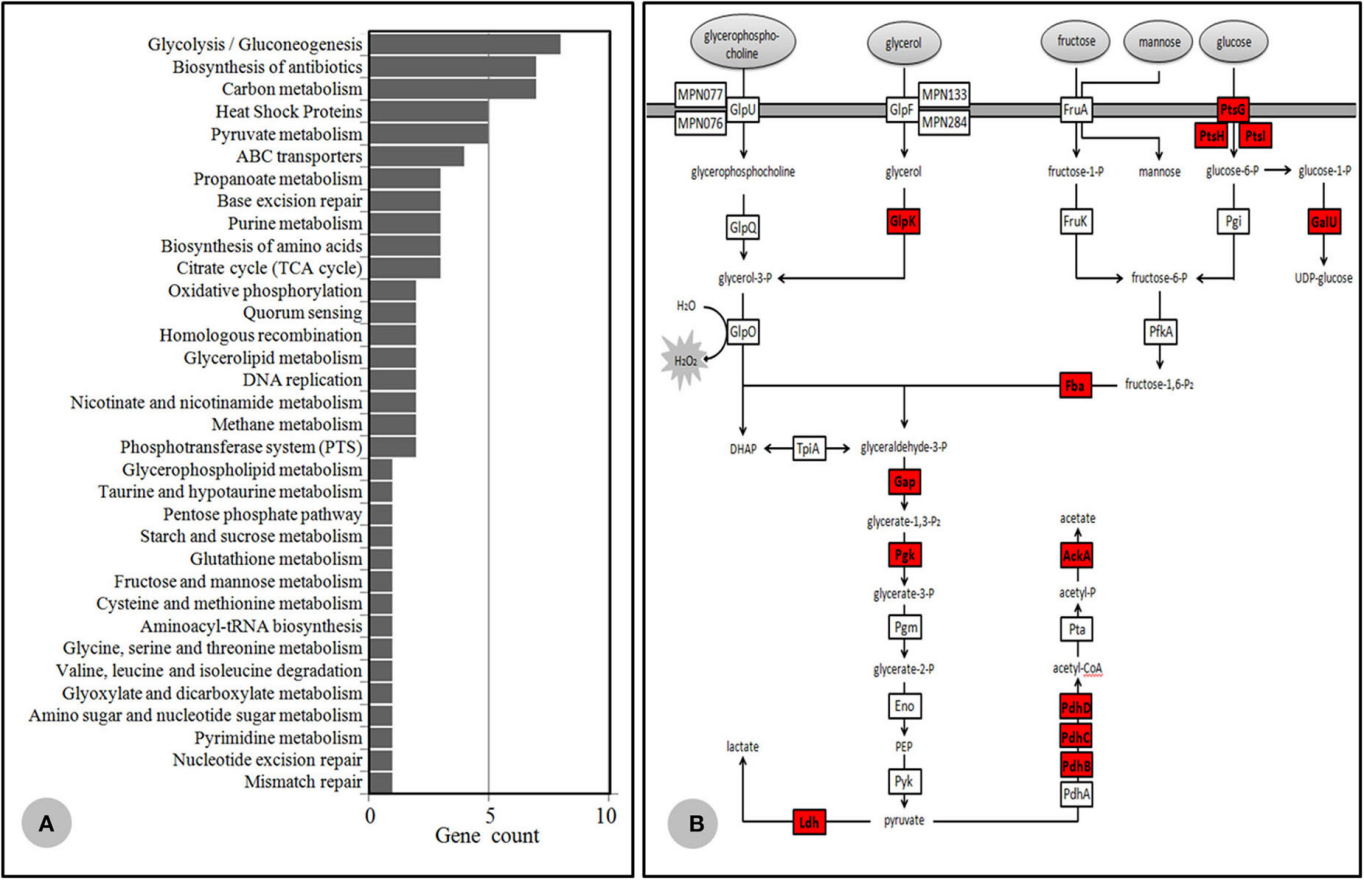
Differential proteomics data. **(A)** KEGG database category findings for the up-regulated protein isolated from MLr *M. pneumoniae*. **(B)** *Mycoplasma pneumoniae* glycometabolism diagram. The generated hydrogen peroxide during the metabolism was important virulence factor for the host cells. The protease highlighted in red color were up-regulated in the MLr *M. pneumoniae*.

Based on TMT quantitation, there were 149 proteins differentially expressed between the Case 35 (MLr, genotype 2) and ICDC P033 (MLs, genotype 2), in which 85 proteins were listed as significantly different with a difference power of >2.0. Meanwhile, 140 proteins were considered to be differentially expressed between ICDC 21109 (MLr, genotype 2) and Case 45 (MLs, genotype 2), in which 68 were listed as significantly different with a power of >2.0. Among these proteins that were significantly expressed, 57 proteins were identified in the 85 proteins and the 68 proteins simultaneously, all of which were up-regulated in the MLr *M. pneumoniae*. More than half of these proteins were membrane-associated proteins KEGG (https://www.genome.jp/kegg/pathway.html) database analysis indicated that 39 proteins were divided into 34 metabolic categories (Figure 4A), while the other 18 proteins were of unknown categories. Among these proteins, most of the proteins were associated with *M. pneumoniae* adhesion and toxicity-related protein, including P1, HMW1, HMW2, P65, MPN142 (P90 and P40), CARDS, PdhB, C and D, ATP synthase subunit beta (AtpD), Elongation factor G and Tu. In addition, another 13 proteins were glycometabolism-related protein in *M. pneumoniae* (Figure 4B), while the other 5 were heat shock proteins (HSPs).

## Discussion

RMPP is a severe disease with a long and complex course affecting the healthy conditions of the children. In this study, we investigated the multiple-pathogen etiology in RMPP patients. Only three RMPP cases (5%) showed bacterial co-infection. The result was close to our previous study (2.67%) (Liu et al., 2018a), and was lower than that of the incidence (15.4%) reported by Zhang et al. (2018) utilizing nasal aspirate samples from RMPP patients. The detected bacteria in that publication mainly included *Streptococcus pneumoniae, Haemophilus influenzae* and *Staphylococcus aureus*. These bacteria can be detected in the pharyngeal portion of the normal individuals, and may be pathogenic under specific conditions, however, we speculated that the BALF samples were more likely to reflect actual infection of the lower respiratory tract. In China mainland, Azithromycin has been frequently utilized for the treatment of *M. pneumoniae* infection in children. This would decrease the incidence of *M. pneumoniae* infection combined with bacterial infection, which may explain for the MPP cases with ML resistance showing low bacterial infection. Compared with bacterial, viral co-infection was more common in RMPP patients. Generally speaking, the pediatric patients of a greater age showed mild infection induced by respiratory syncytial virus or rhinovirus, and their prognosis was usually satisfactory. In addition, some virus-positive patients were asymptomatic at convalescent stage, which suggested that the detected virus might not be responsible for infection. The CRP concentration and the poor prognosis rate in the single *M. pneumoniae* infection RMPP patients both showed no statistical differences compared with that of the RMPP patients with co-infection. This implied that co-infection showed limited effects on the clinical progression and outcome in the RMPP children.

Real-time PCR shows high sensitivity to the early screening of *M. pneumoniae*, but it can not identify the viable strains from the non-viable counterparts. Thus, it was not recommended for the quantification of viable *M. pneumoniae*. PMA combined real-time PCR was utilized for the quantification of the DNA in the viable *M. pneumoniae*. For the same sample, there was significant difference in the total DNA and the viable DNA, which indicated that the number of viable *M. pneumoniae* was far less than that of the conventional DNA determination. There were no viable *M. pneumoniae* in 21 cases, which were both negative with PMA real-time PCR and *M. pneumoniae* culture results, but the patients showed obvious mucus plug in the airway, or deterioration of airway constriction. Thus, bronchovesicular lavage or interventional therapy was still required. We speculated that in the later stage of RMPP, part of the patients may not carry viable *M. pneumoniae*. Nevertheless, certain components (e.g., nucleic acids or polypeptide) may trigger the hyperimmunization, which is the major cause for the persistent airway damage of RMPP and airway occlusion. This implied that more attention should be paid to the anti-inflammation and desensitization rather than the antibacterial therapy in the later stage of RMPP. Among 33 cases that were PMA real-time PCR positive but were *M. pneumoniae* culture negative, 27 (81.8%) showed a viable *M. pneumoniae* count of <10^5^ copies/ml, which was merely higher than that of the culture detection limit of pure *M. pneumoniae* (about 10^3^ copies/ml) (Loens et al., 2002). It seemed reasonable that the viable *M. pneumoniae* with a lower copy number showed negativity by culture, after taking the complexity of clinical samples into consideration. Interestingly, 3 samples (Case 10-2, Case 31-1, and Case 48-1) indicated quantification more than 10^7^copies/ml for viable *M. pneumoniae*, which was >1,000-fold than the culture detection limit of pure *M. pneumoniae*. Despite many attempts to decline the concentration of inhibitors by washing/centrifugation or persistent dilution, the culture results were still negative. We speculate that the *M. pneumoniae* are probably in a state of dormancy to defend against the host immunity that is similar like the viable but non-culturable state (VBNC) in some bacteria exposing to external stimulation (Li et al., 2014; Pienaar et al., 2016). The antimicrobial susceptibility test and the domain V of 23s rRNA sequencing results of the 52 cases indicated that the most typical feature for the *M. pneumoniae* strain identified from the RMPP children was MLr. The total *M. pneumoniae* MLr ratio was in a range of 67% to 100% in China mainland in the past decade. Genotype 1 *M. pneumoniae* showed a high MLr of more than 90%, while that of the genotype 2 showed a MLr of <10% (Xin et al., 2009; Cao et al., 2010; Liu et al., 2010; Zhao et al., 2012b; Zhou et al., 2014, 2015). Recently, the proportion of genotype 2 strains in Beijing showed a tendency of increase. Accordingly, the MLr rate of genotype 2 strains in CMPP cases increased to about 20% (Zhao et al., 2015, 2019a). In this study, the genotype 2 strains showed a MLr rate of up to 90.9%. The rate was higher than that of the rates in the genotype 2 strains from CMPP patients mentioned above. Patients infected with MLr-*M. pneumoniae* showed longer duration of fever and severe imaging results compared with those infected with MLs-*M. pneumoniae*. Besides, the incidence of extra-pulmonary complications is usually high (Cao et al., 2010; Zhou et al., 2014). In the views of etiology, there might be differences in the pathogenecities between MLr and MLs *M. pneumoniae* strains.

The Nano-HPLC-MS/MS proteome data preliminarily confirmed the hypothesis mentioned above. Among the 57 up-regulated proteins in the MLr *M. pneumoniae* strains, 13 proteins associated with the adhesion and toxicity were obviously higher than those of the MLs strains, including the extensively reported P1 (Krause and Balish, 2001; Seto et al., 2001; Nakane et al., 2011), HMW1/HMW2 (Willby et al., 2004), P65 (Kenri et al., 2004), MPN142 (Widjaja et al., 2015), CARDS (Bose et al., 2014; Becker et al., 2015; Ramasamy et al., 2018), as well as the newly identified moonlight protein related to the adhesion such as PdhB-D (Grundel et al., 2015a), ATP synthase subunit beta (AtpD) (Nuyttens et al., 2010), and Elongation factor G,Tu,Ts (Widjaja et al., 2017). These proteins were localized in the cell membrane of *M. pneumoniae*, which involved in the adhesion to the host cells. Besides, the up-regulation of these proteins with toxicity or antigenicity would lead to immunologic stimulation and cell injury. Phospholipids and derived metabolites such as glycerol are the major sources of carbon and energy for *M. pneumoniae* on lung epithelia. During the metabolism of glycerate-3-phosphate in *M. pneumoniae*, the generated hydrogen peroxide was considered the major virulence factors (Grosshennig et al., 2013; Blotz and Stulke, 2017). In this study, 13 proteases associated with glycometabolism were up-regulated in MLr *M. pneumoniae* (Figure 4B). This indicated that the energy synthesis and metabolism in MLr strains was more active than MLs strains. Then the amount of generated hydrogen peroxide would increase, which led to more severe toxicity to the host cells. Some enzymes, including pyruvate dehydrogenases A-C (PdhA-C), glyceraldehyde-3-phosphate dehydrogenase (GapA), lactate dehydrogenase (Ldh), phosphoglycerate mutase (Pgm), pyruvate kinase (Pyk), and transketolase (Tkt), were approved to be closely involved in the glycometabolism. These enzymes also localized on cell membrane of *M. pneumoniae* with antigenic adhesion, and served as a moonlight protein as it could directly mediate the interaction between *M. pneumoniae* and the hosts (Grundel et al., 2015b). Thus, their up-regulation may increase the immunologic stimulation to the hosts. Moreover, more than half of the HSPs in *M. pneumoniae* were up-regulated in the MLr *M. pneumoniae*. Many publications proved that some of the HSPs were localized on the surface of bacteria, which then mediated the interaction between bacteria and the hosts with antigenicity and toxicity (Barnes et al., 1992; LaVerda et al., 2000). Two of the HSPs in *M. pneumoniae* have been detected in the cell surface, which could interact with the host showing a certain antigenicity (Bencina et al., 2005). This implied that HSP played important roles in the pathogenicity of *M. pneumoniae*.

Since the identification of CARDS toxin, serving as the first virulence factor of *M. pneumoniae* (Kannan and Baseman, 2006), more and more proteins have been approved to the related to the pathogenicity of *M. pneumoniae* including Pdh, ATP synthase, GlpD and Elongation factor. This indicated that insufficient protein encoding by the extremely small genomes may be functionally compensated by massive moonlight protein of *M. pneumoniae*. In this study, many proteins in the MLr strains were up-regulated compared to those of the MLs strains, and were associated with the adhesion and toxicity. This implied that the toxicity of MLr *M. pneumoniae* was superior to that of the MLs *M. pneumoniae*. We speculated that more virulent MLr *M. pneumoniae* may be associated with the pathogenesis of RMPP in children. Indeed, not all the strains induced RMPP in children were ML resistant. This implied that autoimmunity is also an important cause for the pathogenesis of RMPP besides the pathogenic causes. The progression of a certain infectious disease is highly relied on the interaction between pathogens and hosts. In future, deep understanding should be established on the RMPP.

There are some limitations in this study. Firstly, the sample is limited, and there might be bias in the cases and the representative pathogenic bacteria. Secondly, the proteomics data are not adequate and further replication and verification are required to the differentially expressed proteins. Thirdly, we focused on the etiology features of *M. pneumoniae*, without investigating the susceptivity of RMPP in children.

## Conclusions

Pediatric RMPP showed longer disease duration and poor prognosis. Part of them presented viral co-infection, but it showed limited effects on the progression and prognosis of RMPP. Many patients may not carry the viable *M. pneumoniae* in the later stage of RMPP, however, certain components may trigger the airway hyper-immunization and remodeling. More attention should be paid to the anti-inflammation and anti-airway remodeling particularly in RMPP. Therefore, it is urgent to identify new drugs for the treatment of RMPP. The expression of virulence factor in the MLr *M. pneumoniae* was higher than that of the MLs *M. pneumoniae*, which was more common in pediatric RMPP cases.

## Data Availability Statement

The proteomic datasets GENERATED for this study can be found in the iprox (IPX0002479000). PXD No.:PXD021568. ProteomeXchange: http://proteomecentral.proteomexchange.org/cgi/GetDataset?ID=PXD021568. iProX: https://www.iprox.org/page/project.html?id=IPX0002479000. The three 16S rRNA sequences uploaded now can be found on NCBI: https://www.ncbi.nlm.nih.gov/nuccore/MW009699, https://www.ncbi.nlm.nih.gov/nuccore/MW009700, and https://www.ncbi.nlm.nih.gov/nuccore/MW009701.

## Ethics Statement

The studies involving human participants were reviewed and approved by National Institute for Communicable Disease Control and Prevention. The patients/participants provided their written informed consent to participate in this study. Written informed consent was obtained from the individual(s), and minor(s)' legal guardian/next of kin, for the publication of any potentially identifiable images or data included in this article.

## Author Contributions

JZ: study design. FZ, JL, DX, and LL: data collection. JG, JX, HL, and SZ: data analysis. All authors contributed to the article and approved the submitted version.

## Conflict of Interest

The authors declare that the research was conducted in the absence of any commercial or financial relationships that could be construed as a potential conflict of interest.

## Abbreviations

BALF: bronchoalveolar lavage fluid
CAP: community acquired pneumonia
CMPP: common *M. pneumoniae* pneumonia
CRP: C-reactive protein
FA: formic acid
GapA: glyceraldehyde-3-phosphate dehydrogenase
HCD: higher energy collisional dissociation
HSPs: heat shock proteins
ICU: intensive care unit
iTRAQ: isobaric tags for relative and absolute quantification
ITS: Internal Transcribed Spacer
Ldh: lactate dehydrogenase
MALDI-TOF MS: Matrix-assisted laser desorption/ionization time-of-flight mass spectrometry
MICs: Minimum inhibitory concentrations
MLr: macrolide resistant
MLVA: multiple locus variable-number tandem repeat analysis
MPP: *M. pneumoniae* pneumonia
PdhA-C: pyruvate dehydrogenases A to C
Pgm: phosphoglycerate mutase
PMA: propidium monoazide
RMPP: refractory *Mycoplasma pneumoniae* pneumonia
Pyk: pyruvate kinase
Tkt: transketolase
TEAB: triethyl ammonium bicarbonate
TNA: total nucleic acids
UPLC: Ultra Performance Liquid Chromatography
VBNC: viable but non-culturable state
WBCs: white blood cells
ERY: erythromycin
AZM: azithromycin
LVX: levofloxacin
TET: tetracycline.
